# Endoscopic risk factors to inform early detection of gastric cancer after *Helicobacter pylori* eradication: Meta‐analysis and systematic review

**DOI:** 10.1002/deo2.70086

**Published:** 2025-02-26

**Authors:** Masaaki Kodama, Osamu Handa, Mitsushige Sugimoto, Takahiro Kotachi, Masaaki Kobayashi, Susumu Take, Shu Hoteya, Katsuhiro Mabe, Takahisa Murao, Ken Namikawa, Takashi Kawai, Kazunari Murakami, Research committee for the Establishment of Risk Evaluation of Gastric Cancer after H. pylori Eradication in Endoscopic Findings, The Japanese Society for Helicobacter Research, Japan

**Affiliations:** ^1^ Department of Advanced Medical Sciences Faculty of Medicine Oita University Oita Japan; ^2^ Department of Gastroenterology Faculty of Medicine Oita University Oita Japan; ^3^ Department of Gastroenterology and Hepatology Kawasaki Medical School Okayama Japan; ^4^ Division of Genome‐Wide Infectious Diseases Research Center for GLOBAL and LOCAL Infectious Diseases Oita University Oita Japan; ^5^ Department of Gastroenterology Hiroshima University Hospital Hiroshima Japan; ^6^ Division of Gastroenterology, Niigata Cancer Center Hospital Niigata Japan; ^7^ Department of Gastrointestinal Endoscopy Nippon Kokan Fukuyama Hospital Okayama Japan; ^8^ Department of Gastroenterology Toranomon Hospital Tokyo Japan; ^9^ Mabe Goryokaku Gastrointestinal Endoscopy Clinic Hokkaido Japan; ^10^ Department of Health Care Medicine Kawasaki Medical School General Medical Center Okayama Japan; ^11^ Department of Gastroenterology Cancer Institute Hospital, Japanese Foundation for Cancer Research Tokyo Japan; ^12^ Department of Gastroenterological Endoscopy Tokyo Medical University Hospital Tokyo Japan

**Keywords:** atrophic gastritis, eradication, gastric cancer, *Helicobacter pylori*, intestinal metaplasia

## Abstract

**Objectives:**

*Helicobacter pylori* eradication reduces but cannot eliminate the risk of gastric cancer (GC). The prevalence of post‐eradication GC has been rising. Characterization of the endoscopic findings of post‐eradication GC may facilitate its early detection. We performed a meta‐analysis and systematic review to clarify endoscopic risk factors to accelerate the early diagnosis of post‐eradication GC.

**Methods:**

Medline and PubMed were searched for randomized controlled trials, cohort studies, and case‐control studies published in the English‐language medical literature between January 1997 and July 2023. The included articles assessed the correlation between post‐eradication GC and pre‐ and post‐eradication endoscopic findings, and associated post‐eradication GC with gastric atrophy, intestinal metaplasia (IM), map‐like redness, and xanthoma.

**Results:**

A total of 963 articles were retrieved. In these articles, 66 papers were finally included, comprising randomized controlled trials, cohort studies, and case‐control studies. The included articles addressed gastric atrophy (16 studies), IM (eight studies), map‐like redness (six studies), and xanthoma (two studies). Risk ratio (RR) of incident post‐eradication GC was 3.40 (95%confidence interval [95%CI]: 1.98–5.84; *p *< 0.001) in cases of severe atrophy, 5.38 (95%CI: 3.62–8.00) in cases of severe IM, 2.34 (95%CI: 1.16–4.68) in cases with post‐eradication map‐like redness, and 2.75 (95% CI: 1.78–4.26) in cases with xanthoma.

**Conclusions:**

Endoscopic atrophy, IM, and xanthoma observed at pre‐ and post‐eradication time points and post‐eradication map‐like redness were suggested as endoscopic risk factors for post‐eradication GC. Further studies are needed to clarify the risk of post‐eradication GC based on these risk factors.

## INTRODUCTION

Gastric cancer (GC) is the fifth most prevalent cancer worldwide and the fourth leading cause of cancer mortality.^1^, ^2^ GC incidence is highest in Japan, where attributable mortality may reach 40,000 deaths annually. *Helicobacter pylori* infection is the major etiology of GC and is implicated in up to 90% of cases.^3^, ^4^ *H. pylori* eradication reduces GC incidence, but only by 60%–70%.^5^, ^6^, ^7^, ^8^ In addition, GC can occur more than ten years after eradication,^9^ thereby posing a lifelong risk. With increased numbers of patients undergoing *H. pylori* eradication, the prevalence of post‐eradication GC has been rising.^10^

Endoscopic and histologic features of post‐eradication GC differ from those of *H. pylori*‐positive cases.^11^, ^12^ Endoscopic diagnosis is difficult, for example, due to the gastritis‐like appearance of gastric epithelium with low‐grade atypia.^13^, ^14^, ^15^ Consequently, the identification of risk factors of post‐eradication GC is important to accelerate early‐stage diagnosis and treatment. Although multiple studies have reported the characteristics and risk factors of post‐eradication GC^9^, ^11^, ^16^, ^17^, ^18^, ^19^, ^20^ and have demonstrated that *H. pylori* eradication suppresses GC,^8^, ^21^, ^22^, ^23^, ^24^ none have comprehensively analyzed endoscopic findings as risk factors for post‐eradication GC. Consequently, the Research Committee for the Establishment of Risk Evaluation of GC after *H. pylori* Eradication in Endoscopic Findings, established by the Japanese Society for *Helicobacter* Research, performed a systematic review and meta‐analysis to clarify endoscopic risk factors to facilitate the early diagnosis of post‐eradication GC.

## MATERIALS AND METHODS

### Search strategy and selection criteria

We searched the PubMed and Medline registries according to the Preferred Reporting Items for Systematic Reviews and Meta‐Analysis (PRISMA) guidelines (PRISMA 2020 Checklist in Supporting Information). The search was conducted using the following terms: ‘Stomach Neoplasms’ [MeSH Major Topic], ‘pylori,’ and ‘eradication,’ and ‘(after or following or post or past).’ Inclusion criteria were (1) evaluation of the association between *H. pylori* eradication and post‐eradication GC; and (2) evaluation of the correlation of endoscopic findings to post‐eradication GC. Included study designs comprised randomized controlled trials (RCTs), prospective cohort studies, retrospective cohort studies, and case‐control studies published in the English‐language medical literature from January 1993 to July 2023.

### Study design and data collection

Eight investigators of the study committee (Masaaki Kodama, Osamu Handa, Mitsushige Sugimoto, Takahiro Kotachi, Masaaki Kobayashi, Susumu Take, Syu Hoteya, and Takahisa Murao) each independently screened titles and abstracts and then evaluated the full texts of the selected articles. Case reports and review articles, studies regarding gastric lesions other than post‐eradication GC (e.g., mucosa‐associated lymphoid tissue lymphoma, adenoma, and hyperplastic polyps; dysplasia; gastric ulcer; GC but not after eradication) were excluded. Basic research articles and clinical studies without assessments of risk factors or endoscopic findings were excluded. All articles that were considered necessary but not identified by this search strategy were hand‐searched for inclusion. Finally, committee members discussed and determined the validity of the collected literature.

### Outcomes

The primary outcome was the development of post‐eradication GC. The period from eradication to GC diagnosis was recognized as more than 1 year but followed the case definitions of the respective studies. Asymptomatic cases in healthy subjects or first GC development after *H. pylori* eradication indicated for the treatment of benign diseases such as peptic ulcer were defined as cases without GC at baseline. Metachronous GC after endoscopic resection of early GC was defined as cases with GC at baseline. Baseline factors, comorbidities, endoscopic findings, histologic findings, and medications that were not analyzed and correlated with post‐eradication GC were excluded.

The secondary outcome was the relationship of specific endoscopic findings to incident post‐eradication GC. Endoscopic findings associated with post‐eradication GC in the collected articles included atrophy, intestinal metaplasia (IM), map‐like redness, and xanthoma.

Atrophy comprises both histologic and endoscopic atrophy. Histologic atrophy was defined in enrolled studies by the updated Sydney system using a visual analog scale^25^ and was classified as normal, mild, moderate, and marked. Endoscopic atrophy was defined by the Kimura‐Takemoto classification, that is, close‐type and open‐type atrophy were further classified as: none (C‐0), mild (C‐1, C‐2), moderate (C‐3, O‐1), and severe (O‐2, O‐3).^26^ Included articles also addressed endoscopic findings of IM in the antrum and the corpus; the onset of map‐like redness after *H. pylori* eradication^27^; and the presence of xanthomas.

Atrophy due to *H. pylori* infection reduces the acidity of the gastric environment, which induces IM at atrophied sites. Therefore, atrophy and IM are strongly correlated. Narrow band imaging (Olympus) and Blue laser imaging (Fujifilm) disclose light blue crests and a white opaque substance as characteristic findings on the IM surface layer.^28^, ^29^

Most endoscopic findings associated with *H. pylori* infection, such as petechial and diffuse erythema, accompany current infection but often decrease or resolve after eradication. Map‐like redness appears after eradication and persists thereafter.^27^ Map‐like redness consists of depressed and variously shaped erythema formed by the difference in change between IM and the surrounding mucosa after successful eradication. Not all IM lesions develop post‐eradication map‐like redness; incidence is 20%–30% one year after eradication.^30^, ^31^

### Statistical analysis

Heterogeneity was evaluated by *I* ^2^ value and Cochran's Q. The *I* ^2^ value was used to assess the heterogeneity of the studies as follows: 0%–39%, low heterogeneity; 40%–74%, moderate heterogeneity; and 75%–100%, high heterogeneity. The risk ratio (RR) and 95% confidence interval (CI) of each study were reported as the measure of effect size. Mainly random‐effects model was used for the evaluation of each model, in addition, the fixed‐effects model was also calculated. Publication bias was assessed using funnel plots. All meta‐analyses were conducted using open‐source statistical software (Review Manager Version 5.3. Copenhagen: The Nordic Cochrane Centre, the Cochrane Collaboration, 2014). All *p‐*values were two‐sided, and *p* < 0.05 was considered statistically significant.

## RESULTS

### Data extraction

**FIGURE 1:**
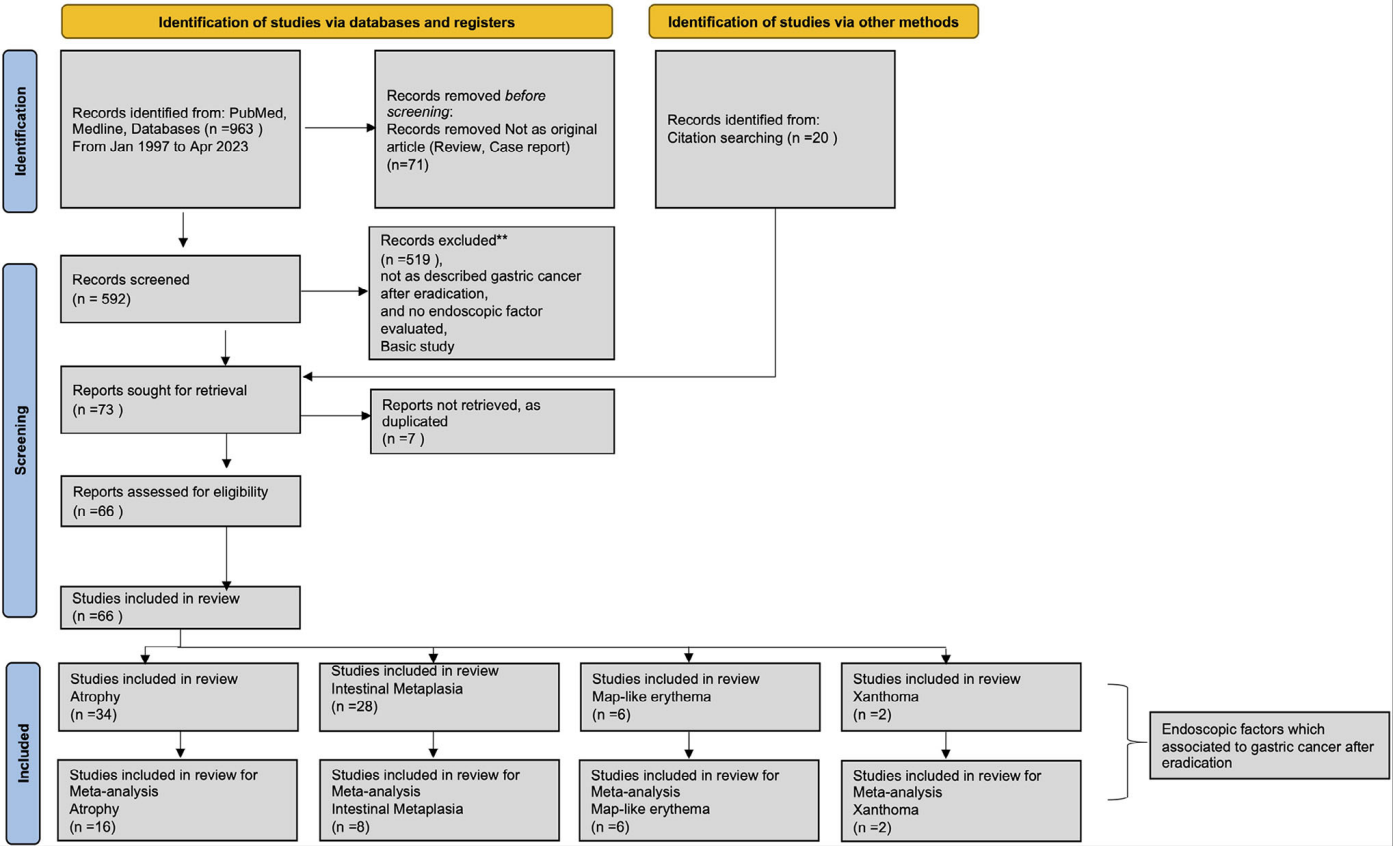
Flow diagram of search and study selection. Flow diagram of search and study selection according to Preferred Reporting Items for Systematic Reviews and Meta‐Analyses (PRISMA) guidelines (PRISMA 2020 Checklist in Supporting information). The included articles addressed gastric atrophy (16 studies), intestinal metaplasia (eight studies), map‐like redness (six studies), and xanthoma (two studies).

A total of 963 articles were retrieved. Articles that did not report original research (review articles, case reports; n = 71); that did not describe post‐eradication GC; that did not evaluate endoscopic findings; or that reported basic research (n = 519) were excluded. A total of 66 papers were finally included, comprising RCTs, cohort studies, and case‐control studies. Four endoscopic findings comprising atrophy, IM, map‐like redness, and xanthoma were analyzed in these articles as factors associated with post‐eradication GC. Therefore, articles were selected and analyzed based on these four endoscopic findings (Figure 1).

### Meta‐analysis

Of the 66 collected articles, 34 addressed atrophy, 28 evaluated IM, six described map‐like redness, and two that evaluated xanthomas were selected as references that analyzed relationships between endoscopic findings and post‐eradication GC. Of these, 15 articles on atrophy, eight on IM, six on map‐like redness, and two on xanthoma were finally selected for meta‐analysis. Overall, 19 articles were analyzed after duplicates were excluded.

### Atrophy

**FIGURE 2:**
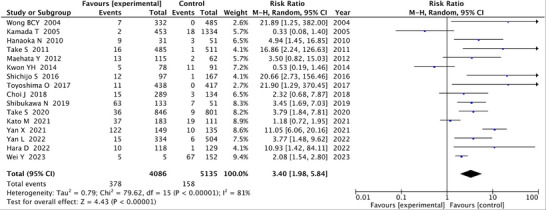
Forest plot analyzing the occurrence of post‐eradication gastric cancer according to severity of gastric atrophy.

Five of the 16 articles subjected to meta‐analysis evaluated histologic atrophy and 11 analyzed endoscopic atrophy. Nine articles studied cases with pre‐eradication GC, and seven articles analyzed cases without GC at baseline. Definitions of severe, mild, or no atrophy differed between articles, with some articles classifying only by the presence of atrophy and others using the Kimura‐Takemoto classification, with O‐2 and O‐3 signifying severe and C0‐C2 representing absent or mild atrophy (Table 1). A total of 378 of 4086 patients with severe atrophy developed post‐eradication GC, compared to 158 of 5135 cases with no or mild atrophy. The corresponding forest plot is shown in Figure 2. The overall RR for the onset of post‐eradication GC among patients with severe atrophy was 3.40 (95%CI: 1.98–5.84; *p *< 0.001) compared to no or mild atrophy. The evaluation of heterogeneity disclosed an *I* ^2^ of 81% (Figure 2), (Fixed‐effects model is shown in Figure S1).

**TABLE 1:** Baseline characteristics of studies that indicated a correlation between atrophy and post‐eradication gastric cancer.

Author	Year	Country	Study Design	Baseline GC	Atrophy, histological, or endoscopic	Sample size (Eradication cases)	GC cases after eradication	Risk ratio	95％ CI	*p‐*value	Comment
Wong BCY^36^	2004	China	RCT	Without GC	Histological	817	18	HR: 2.97	0.94–9.42	0.06	Precancerous lesion (histological atrophy, IM, dysplasia) HR: 2.97 (0.94–9.42) .06
Kamada T^63^	2005	Japan	Cohort	Without GC	Histological	1787	20	NA			GC/Atrophic gastritis: 2/453(0.4％)
Hanaoka N^67^	2010	Japan	Cohort	With GC	Endoscopic	82	12	HR: 4.88	1.32–18.2	0.018	Open‐type atrophy diagnosed by AFI was significantly associated with metachronous GC (HR: 4.88, 95%CI: 1.32–18.2, *p* = 0.018)
Take S^46^	2011	Japan	Cohort	Without GC	Endoscopic	1674	28	HR: 14.4	1.9–110.2	0.01	GC group: Severe atrophy at Baseline (HR: 14.4;95％CI: 1.9–110.2, *p*=0 .01 vs. mild atrophy)
Maehata Y^68^	2012	Japan	R Cohort	With GC	Endoscopic	177	15	OR: 2.71	1.07–7.97	0.036	Severe atrophy; OR: 2.71 (1.07–7.97) *p* = 0.036
Kwon YH^64^	2014	Korea	R Cohort	With GC	Endoscopic	214	18	NA			Risk of GC is HP infection OR 2.322, Age 60 years old ≧(OR: 2.803), Endoscopic atrophy showed no significance
Shichijo S^55^	2016	Japan	Cohort	Without GC	Histological	573	21	HR: 9.3	1.7–174	0.007	Severe atrophy (O2‐O3): 12 of 97 cases HR: 9.3 95%CI: 1.7–174, *p* = 0.007 versus. non‐mild Atrophy (C0‐C2)
Toyoshima O^65^	2017	Japan	R Cohort	Without GC	Endoscopic	1232	15	HR: 1.7	1.12–2.78	0.01	Atrophy;(HR: 1.7, 1.12–2.78 0.01)
Choi JM ^33^	2018	Korea	RCT	With GC	Histological	442	18	NA			GC incidence of no‐Corpus Atrophy 3/134, corpus atrophy 15/289
Shibukawa N^41^	2019	Japan	Case Control	With GC	Endoscopic	184	70	OR: 3.06	1.10–8.49	0.03	Atrophy (open type): OR 3.06 (1.10–8.49) 0.03
Take S^69^	2020	Japan	R Cohort	Without GC	Endoscopic	2737	68	NA			GC incidence of severe atrophy 0.67%/year, versus moderate 0.29%/year, mild atrophy 0.15%/year
Kato M^66^	2021	Japan	Case Control	With GC	Endoscopic	294	52	NA			HR: 1.20 times higher in the non‐eradicated group than in the eradicated group in the advanced atrophy group (*p* = 0.52)
Yan X^40^	2021	China	R Cohort	With GC	Endoscopic	1961	132	OR: 8.08	3.43–20.0	<0.01	Severe atrophy: (OR = 8.08; 95% CI, 3.43‐20.0; *p* < 0.01)
Hara D^42^	2022	Japan	Case Control	Without GC	Endoscopic	247	11	NA			atrophy close type GC 1/11, open type GC10/11
Yan L^32^	2022	China	RCT	Without GC	Histological	817	21	HR: 0.37	0.15–0.95		Without precancerous lesion (histological atrophy, IM, dysplasia), HR: 0.37; 95% CI, 0.15–0.95
Wei Y^10^	2023	China	Case Control	With GC	Endoscopic	133	133	NA			Moderate/severe gastric atrophy, IM of the gastric body, severe diffuse erythema, and map‐like erythema are risk factors for EGC after eradication

Abbreviations: 95%CI: 95% Confidence Interval, GC: gastric cancer, HP: *Helicobacter pylori*, HR: hazard ratio, NA: not applicable, OR: odds ratio, RCT: randomized controlled trial, R cohort: retrospective cohort.

### Atrophy, sub‐analysis with and without GC at baseline

**FIGURE 3:**
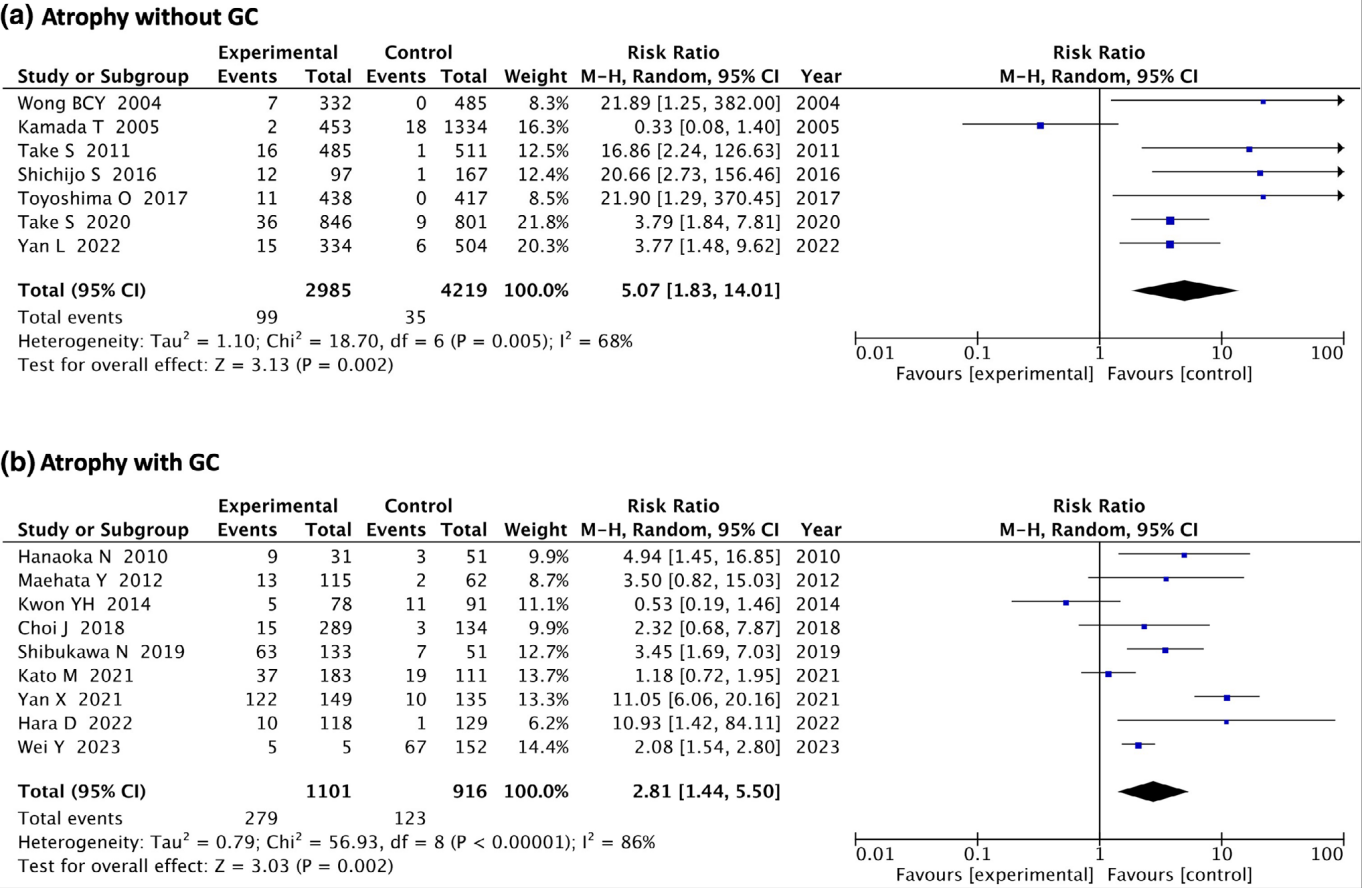
Forest plot analyzing the occurrence of post‐eradication gastric cancer according to the severity of gastric atrophy, Sub‐analysis with and without GC at baseline. (a) Forest plot of Atrophy without GC. (b) Forest plot of Atrophy with GC. GC: gastric cancer.

Due to the high heterogeneity in atrophy (81%), we performed sub‐analysis by a group of seven articles without GC and nine articles with GC (after endoscopic resection of GC) in the baseline as sensitivity analysis (Figure 3), (Fixed‐effects model is shown in Figure S2).

In the without GC group, RR of severe atrophy showed 5.07 (95%CI: 1.83–14.01; *p* = 0.005) compared to no or mild atrophy. Heterogeneity was 68% (Figure 3a). With the GC group, severe atrophy showed RR 2.81 (95%CI: 1.44–5.50; *p* < 0.001) compared to no or mild atrophy. Heterogeneity was 86% (Figure 3b).

### Intestinal metaplasia

**FIGURE 4:**
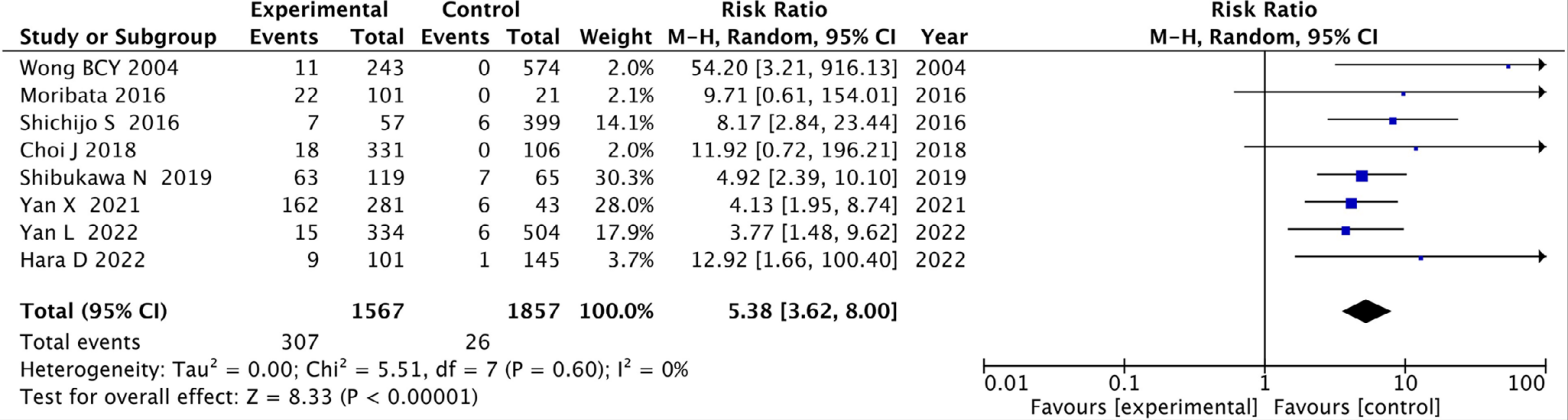
Forest plot analyzing the occurrence of post‐eradication gastric cancer according to severity of intestinal metaplasia. IM: intestinal metaplasia.

Of the eight articles addressing IM that were subjected to meta‐analysis, three described endoscopic evaluation, and five reported histologic assessment (Table 2). Criteria for determining the degree of IM were the presence or absence of IM, or the presence of IM limited to the antrum or extending to the corpus. In most articles, IM of the corpus was classified as severe IM. Post‐eradication GC was diagnosed in 307 of 1567 patients with severe IM and in 26 of 1857 patients without or with mild IM. RR of post‐eradication GC among patients with advanced IM was 5.38 (95%CI: 3.62–8.00) compared to no or limited to the antrum (Figure 4), (Fixe‐effects model is shown in Figure S3). Heterogeneity was low, with an *I* ^2^ value of 0% (Table 3).

**TABLE 2:** Baseline characteristics of studies that correlated intestinal metaplasia with post‐eradication gastric cancer.

Author	Year	Country	Study design	Baseline GC	IM, histologic, or endoscopic	Eradication cases	GC cases after eradication	Risk ratio	95％ CI	*p‐*Value	Comment
Wong BCY^36^	2004	China	RCT	−	Histologic	817	18	HR 2.97	0.94‐9.42	0.06	Precancerous lesion (histological atrophy, IM, dysplasia), HR: 2.97 (0.94–9.42), *p* = 0.06
Moribata K^30^	2016	Japan	R. Cohort	+	Histologic	122	22	NA			Kaplan–Meier curves indicated that patients without IM before ESD never developed metachronous cancer.
Shichijo S^55^	2016	Japan	Cohort	−	Histologic	573	21	HR 3.7	1.1‐12		IM in the corpus: HR 3.7(95％CI, 1.1–12)versus non‐IM
Choi JM^56^	2018	Korea	RCT	+	Histologic	442	18	NA			Corpus IM 18/331, No corpus IM 0/106
Shibukawa N^41^	2019	Japan	Case control	+	Endoscopic	184	70	OR: 4.39	1.58‐12.2	0.005	IM: OR 4.39 (1.58–12.2), *p* = 0.005
Yan X^40^	2021	China	R. Cohort	+	Endoscopic	1961	132	OR: 2.16	0.51‐11.8	0.32	IM: (OR = 2.16; 95% CI, 0.51–11.8; *p* = 0.32)
Hara D^42^	2022	Japan	Case control	−	Endoscopic	247	11	NA			OLGIM I/II: GC 1/11, OLGIM III/IV: GC10/11
Yan L^32^	2022	China	RCT	−	Histologic	817	21	HR: 0.37	0.15–0.95		Without precancerous lesion (histological atrophy, IM, dysplasia) (HR: 0.37; 95% CI, 0.15–0.95)

Abbreviations: 95%CI: 95% confidence interval, HR: hazard ratio, IM: intestinal metaplasia, NA: not applicable, OLGIM: operative link on gastric intestinal metaplasia assessment, OR: odds ratio, RCT: randomized controlled trial, R cohort: retrospective cohort.

**TABLE 3:** Baseline characteristics of studies that correlated map‐like redness and post‐eradication gastric cancer.

Author	Year	Country	Study design	Baseline GC	Number of cases	Emergence of map‐like redness (%)	OR	95% CI	*p‐*value	Comments
Moribata K^30^	2017	Japan	R. Cohort	+	122	32 (39/122)	3.61	1.41–9.21	0.007	Map‐like redness in 64% (14/22) of patients who developed metachronous GC after eradication and in 25% (25/100) of those who did not develop metachronous GC.
Majima A^60^	2019	Japan	Case control	+	109	WLI: 61.5 (67/109) LCI: 78 (85/109)	2.05 (WLI) 3.62 (LCI)	1.09–3.87 1.88–6.97	0.03 0.0001	With LCI observation, map‐like redness was significantly higher in the GC after‐eradication group (45.9%) than in the non‐GC group (78%).
Ohno A^70^	2020	Japan	Case control	Both + and −	43	60.5 (26/43)	NA			Map‐like redness was significantly higher in post eradication GC group (60.5%) than in the non‐GC group (31.1%).
Yan X^40^	2021	China	R. Cohort	+	162	89.5 (145/162)	1.75	1.11–5.25	0.04	Map‐like redness was significantly higher in post eradication GC group (89.5%) than in the non‐GC group (65.4%).
Liu X^71^	2023	China	Case control	+	81	85.2 (69/81)	32.18	9.26–111.77	<0.001	Map‐like redness was significantly higher in post eradication GC group (85.2%, 69/81) than in the non‐GC group (13.3%, 14/105).
Wei Y^10^	2023	China	R. Cohort	+	133	41 (55/133)	1.21	0.55–2.64	0.632	Map‐like redness was significantly higher in post eradication GC group (41%) than in the non‐GC group (24%).

Abbreviations: 95%CI: 95% confidence interval, GC: gastric cancer, HR: hazard ratio, LCI: linked color imaging, NA: not applicable, OR: odds ratio, RCT: randomized controlled trial, R cohort: retrospective cohort, WLI: white light imaging.

### Map‐like redness

**FIGURE 5:**
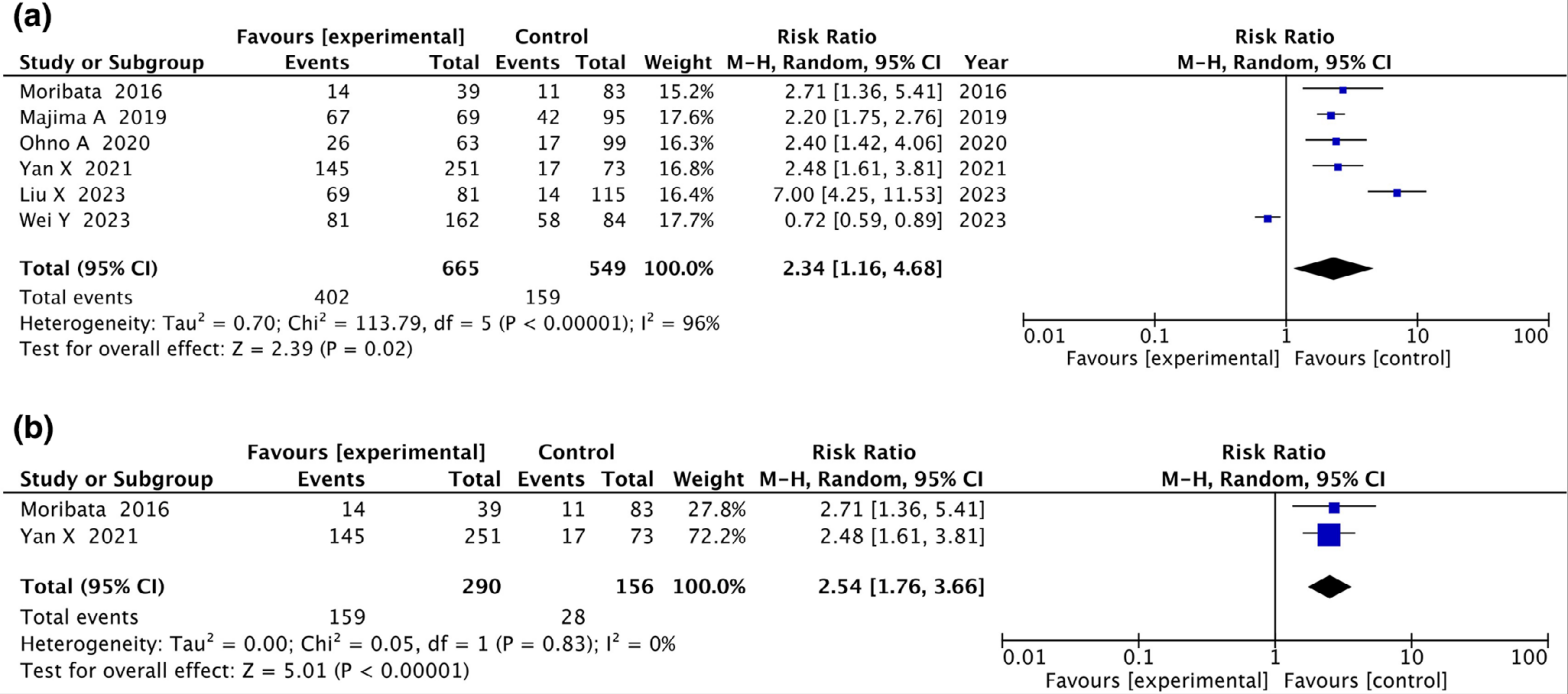
Forest plot analyzing the occurrence of post‐eradication gastric cancer according to post‐eradication onset of map‐like redness. (a) Forest plot analyzed using all articles. (b) Forest plot analyzed using selected two prospective cohort studies.

The six articles that associated post‐eradication GC with map‐like redness were subjected to meta‐analysis (Table 5) A fixed‐effects model is shown in Figure S4). Because map‐like redness is a gross endoscopic finding, no histologic analyses were performed. A total of 402 of 665 patients with post‐eradication map‐like redness developed post‐eradication GC, compared with 159 of 549 patients without map‐like redness who developed post‐eradication GC. RR was 2.34 (95%CI: 1.16–4.68) compared to no detection of map‐like redness. Heterogeneity was high, with an *I* ^2^ value of 96% (Figure 5a).

Map‐like redness had high heterogeneity (96%), therefore, subanalysis with only two articles of prospective cohort studies was performed. In this analysis, the RR was 2.54 (95%CI: 1.76–3.66; *p *= 0.83) compared to non‐Map‐like redness (Figure 5b).

### Xanthoma

**FIGURE 6:**
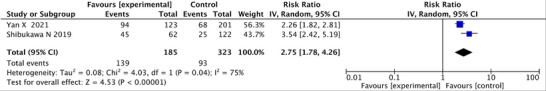
Forest plot analyzing the incidence of post‐eradication gastric cancer according to the appearance of xanthoma.

**FIGURE 7:**
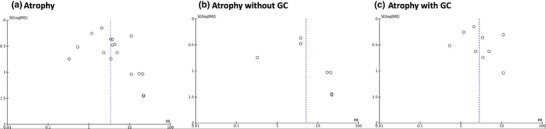
Funnel plots of atrophy analyzed. (a) Atrophy of all 16 articles, (b) atrophy of seven articles without gastric cancer (GC), and (c) atrophy of nine articles with GC.

**FIGURE 8:**
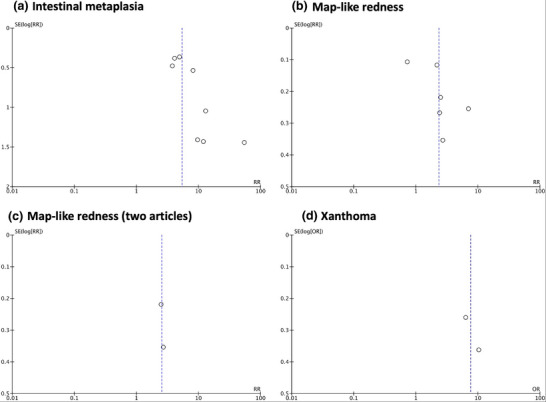
Funnel plots of intestinal metaplasia, map‐like redness, and xanthoma analyzed. (a) Intestinal metaplasia, (b) map‐like redness of all six articles, (c) map‐like redness of selected two articles, and (d) xanthoma.

Both articles that associated post‐eradication GC and xanthoma confirmed the presence of xanthoma prior to *H. pylori* eradication and evaluated post‐eradication GC after endoscopic resection of early‐stage GC (Table 4). Post‐eradication GC developed in 139 of 185 patients with xanthoma, compared with 93 of 323 patients without xanthoma. RR of developing post‐eradication GC among patients with xanthoma was 2.75 (95% CI: 1.78–4.26) compared to no presence of xanthoma (Figure 6); a fixed‐effects model is shown in Figure S5. Heterogeneity was high, with an I^2^ value of 75%. Publication bias, which was assessed by funnel plots, are shown in Figure 7 (atrophy) and Figure 8 (intestinal metaplasia, map‐like redness, and xanthoma). Funnel plots using a fixed‐effects model are shown in Figures S6 and S7.

**TABLE 4:** Baseline characteristics of studies that correlated xanthoma and post‐eradication gastric cancer.

Author	Year	Country	Study design	Baseline GC	Xanthoma, histological, or endoscopic	Eradication cases	GC cases after eradication	Risk ratio	95％CI	*p*‐value	Comment
Shibukawa N^41^	2019	Japan	Case control	+	Endoscopic	184	70	OR: 5.64	2.47–12.9	<0.0001	Xanthoma: OR 5.64 (2.47–12.9), *p* < 0.0001
Yan X^40^	2021	China	R. Cohort	+	Endoscopic	1961	132	OR: 2.84	1.20–7.03	0.02	Xanthoma: (OR 2.84; 95% CI, 1.20–7.03; *p* = 0.02)

Abbreviations: 95%CI: 95% confidence interval, OR: odds ratio, R cohort: retrospective cohort.

### GC development with and without GC before eradication

In addition, evaluation of the number of post‐eradication GC in five with‐GC and seven without‐GC, which were all cohort and RCT articles, was demonstrated. GC group showed 205 of 2916 (7.03%) and without‐GC group showed 191 of 9673 (3.15%). The with‐GC group showed a significantly higher incidence of post‐eradication GC than the without‐GC group (*p* < 0.001; Table 5).

**TABLE 5:** Gastric cancer (GC) development with or without GC before eradication.

		GC	Total	*p*‐value
GC development with GC before eradication	Five articles	205 (7.03%)	2916	<0.001
GC development without GC before eradication	Seven articles	191 (1.98%)	9673	
Total	12 articles	396 (3.15%)	12,586	

GC: gastric cancer, Statistical analysis was used chi‐square test.

## DISCUSSION

An association between *H. pylori* eradication and GC has been disclosed by RCTs,^6^, ^32^, ^33^ cohort studies,^9^ and meta‐analyses.^22^, ^34^ Risk factors of post‐eradication GC have also been evaluated. Sugano reported in his meta‐analysis that the incidence of GC is significantly lower after eradication (odds ratio [OR] 0.46; 95％CI: 0.39–0.55).^23^ However, to our knowledge, meta‐analysis and systematic review of endoscopic findings associated with the risk of post‐eradication GC has not been performed. Among the 66 articles selected for this study, four endoscopic findings were considered risk factors of post‐eradication GC: atrophy, IM, map‐like redness, and xanthoma. Other factors such as age, sex, medical history, and histologically confirmed inflammation were also reported previously as risk factors of post‐eradication GC,^18^, ^35^, ^36^, ^37^, ^38^, ^39^ but were removed in this study except for factors related to endoscopic findings.

Overall, 19 papers were subjected to meta‐analysis after the exclusion of duplicates. Yan et al. identified all four factors as risk factors,^40^ while Shibukawa et al. showed atrophy, IM, and map‐like redness as risk factors.^41^ Five articles analyzed both atrophy and IM as risk factors.^32^, ^33^, ^36^, ^42^, ^43^ This result also indicated the close involvement of atrophy, IM, and map‐like redness in *H. pylori*‐infected gastric mucosa.

Classified by country, 12 articles were from Japan, six from China, and one from South Korea. In the meta‐analysis by Lee et al.,^22^ most articles were from East Asia (12 from Japan, four from China, one from Taiwan, and five from South Korea), with only one from Colombia and one from Finland. East Asian countries such as Japan, South Korea, and China account for about 70% of global GC incidence and mortality.^1^, ^2^ Geographic differences in GC prevalence suggest increased carcinogenicity of East Asian variants of *H. pylori* virulence factors such as CagA.^44^, ^45^ Therefore, *H. pylori* treatment and endoscopic screening for GC must be considered in East Asian countries.

### Atrophy

Meta‐analysis of the ten articles on endoscopic atrophy and five on histologic atrophy yielded conflicting results but disclosed a 4‐fold risk of post‐eradication GC among patients with severe atrophy compared to those with absent or mild atrophy. Take et al. reported a significant risk of post‐eradication GC for patients with endoscopic severe atrophy, with HR 14.4, 95% CI: 1.9–110.2 (*p* = 0.01) for baseline severe versus mild atrophy in 1674 successfully eradicated peptic ulcer cases in a prospective cohort study.^46^ Although severe atrophy extending to the corpus was associated with a high risk of *H. pylori*‐infected GC previously,^3^ the present study showed that open‐type severe endoscopic atrophy extending to the corpus was a significant risk factor for both pre‐ and post‐eradication GC. The high heterogeneity (81%) observed in our study may be attributed to a mixture of endoscopic and histologic atrophy and differences in classifying the degree of endoscopic atrophy.

In the present study, the rate of metachronous GC after GC resection was significantly higher than that of GC without GC history. Sugano reported in his meta‐analysis that the GC incidence in the group with GC at baseline (5.26%) was higher than without GC (1.13%). Fukase also indicated that the post‐eradication GC incidence after GC resection (3.5%) was higher than that without eradication (9.6%). The present study showed similar GC incidence with or without GC history. However, sub‐analysis of both GC with and without GC at baseline showed significantly higher relative risks for severe atrophy. This suggests that atrophy is a major risk factor for GC after eradication, despite differences between with and without GC at eradication.

The Kimura‐Takemoto classification scheme is a globally accepted system that indicates the border of endoscopic atrophy, that is, the border between the fundic gland region and areas of metaplasia and/or pyloric glands.^26^ Although this classification does not directly indicate the extent of histologically confirmed gastric atrophy, a significant correlation between endoscopic and histologically confirmed atrophy and IM of both the antrum and the corpus has been reported.^47^, ^48^, ^49^ Therefore, histologic and endoscopic atrophy were considered to be equivalent.

After eradication, histologic atrophy improves gradually,^50^, ^51^, ^52^, ^53^ while endoscopic atrophy is persistent.^54^ Most studies of the risk of post‐eradication GC have evaluated pre‐eradication baseline endoscopic atrophy. However, its negligible improvement after eradication suggests that advanced endoscopic atrophy may represent a high risk of incident GC at any post‐eradication time point. With the increasing number of cases with either an unknown history of eradication or spontaneous eradication, risk assessment‐based endoscopic atrophy is important at any time point after eradication.

### Intestinal metaplasia

This study included five articles on histologic^30^, ^32^, ^36^, ^55^, ^56^ and three articles on endoscopic IM.^40^, ^41^, ^42^ According to the Kyoto classification of gastritis,^27^ advanced IM extends beyond the vestibule to the corpus. Cases with IM extending into the corpus showed a nearly 9‐fold higher risk than the group without IM or with mild antral IM.

Unlike histologic atrophy, histologic IM is recalcitrant after eradication,^50^ as confirmed by three meta‐analyses.^52^, ^57^, ^58^ Therefore, the extent of IM before eradication and also at any post‐eradication time point correlates with post‐eradication GC. Shichijo et al. evaluated the distribution of endoscopic IM in three groups: no IM, IM confined to the antrum, and extension to the corpus,^55^ and found that advanced IM increased the risk of post‐eradication GC. Multivariate analysis in our previous study also identified histologic IM of the corpus as the highest risk factor for post‐eradication GC.^18^

Takeuchi et al. reported that IM is a precancerous gastric lesion based on DNA methylation profiles.^59^ These epigenetic abnormalities, as well as atrophy, may be considered as mechanisms by which IM increases GC risk.

### Map‐like redness

In the present meta‐analysis, six articles on map‐like redness found that map‐like redness approximately doubled the risk of post‐eradication GC. We found no RCTs or prospective cohort studies on map‐like redness.

Because of the high heterogeneity of the analysis of Map‐like redness, a sub‐analysis of two prospective cohort studies was performed for sensitivity analysis. The RR was 2.54 in this analysis, suggesting that map‐like redness may be a useful risk predictor. However, the number of papers analyzed was only two, and further study is needed.

Moribata et al. reported that post‐eradication map‐like redness correlated significantly with metachronous GC development.^30^ Majima et al. found that endoscopic screening with linked color imaging (Fujifilm Medical Co. Ltd.) exhibited a higher recognition rate of map‐like redness than with observation under white light.^60^

### Xanthoma

Gastric xanthomas are white to yellowish sessile lesions with a fine granular surface found in *H. pylori*‐infected gastric mucosa. Histologic characteristics include aggregations of lipid‐phagocytosing histiocytes in the lamina propria. The morphology of xanthomas persists after *H. pylori* eradication. In the present study, both articles on the association between xanthoma and the risk of post‐eradication GC were retrospective studies^40^, ^41^; however, gastric xanthoma carried an approximately 7‐fold risk of post‐eradication GC. The multivariate logistic regression analysis conducted by Shibukawa et al. identified gastric xanthoma as an independent predictor of early post‐eradication GC (OR 5.64, 95% CI 2.47–12.9).^41^

Many of the endoscopic findings that accompany active *H. pylori* infection improve or resolve after eradication. However, GC may occur more than 10 years after eradication. In addition, the number of cases with no known history of eradication is increasing.^61^, ^62^ Many studies included in our analysis evaluated endoscopic atrophy, IM, and xanthoma before eradication. However, all these endoscopic findings persisted after eradication. These results suggest that even cases that occur more than 10 years after eradication or cases with an unknown history of eradication may be useful in assessing the risk of post‐eradication GC.

Eradication cannot completely inhibit GC. Take et al. found GC 18.3 years after eradication.^61^ Kobayashi et al. reported that invasive cancers diagnosed over ten years after eradication were likely to be more malignant in histological type and pathological stage.^62^ In our previous study, absent endoscopic follow‐up was the risk of post‐eradication undifferentiated GC. ^20^ Therefore, long‐term endoscopic screening must be performed in all cases, even after eradication. However, the indicators are necessary to improve endoscopic screening efficiency.

Many studies indicated that severe atrophy, IM, and Map‐like redness are associated with the risk of GC after eradication. However, there have been no reports using meta‐analysis or systematic review of endoscopic factors associated with the risk of post‐eradication GC. The strength of our study is that endoscopic risk factors and findings for GC diagnosis after eradication were confirmed by meta‐analysis for the first time.

Screening focusing on these factors may improve the accuracy of diagnosis of GC after eradication. In addition, further studies, including a large number of cases, may lead to the discovery of new screening methods and endoscopic risk factors.

### Limitations

Several limitations of this study must be acknowledged. First, the definitions of the degrees of atrophy and IM differed between studies. Histological and endoscopic evaluations were also examined together. Although the histological and endoscopic findings were correlated, separate analyses were considered necessary. In addition, studies that indicated O‐2 and O‐3 as severe atrophy according to the Kimura‐Takemoto classification and studies that rated atrophy only by its presence or absence were included, and no unanimous criteria were used.

Second, because of the selection of studies focusing on endoscopic findings related to post‐eradication GC, many retrospective case‐control studies were also included. Prospective studies of endoscopic findings observed in the present study will be necessary.

Third, in the funnel plot, some partial blanks were observed in the analysis of atrophy. This result is considered a possible publication bias. Because GC often develops from severe atrophy, the possibility that few studies correspond to the blank area of the funnel plot may be considered. Further accumulation of articles is needed.

In conclusion, we conducted a meta‐analysis and systematic review to extract endoscopic risks of post‐eradication GC. Endoscopic atrophy, IM, and xanthoma observed at pre‐ and post‐eradication time points, and post‐eradication map‐like redness were suggested as endoscopic risk factors for post‐eradication GC. These results suggest the need for post‐eradication endoscopic screening and continued GC risk assessment with attention to these findings.

## CONFLICT OF INTEREST STATEMENT

None.

## PATIENT CONSENT STATEMENT

All procedures were performed in accordance with the ethical standards of the institutional review board of the Oita University Faculty of Medicine and with the Helsinki Declaration of 1964 and later versions.

## Supporting information




**FIGURE S1** Forest plot analyzing the occurrence of post‐eradication gastric cancer according to the severity of gastric atrophy, using a fixed‐effect model.

**FIGURE S2** Forest plot analyzing the occurrence of post‐eradication gastric cancer according to the severity of gastric atrophy, Sub‐analysis with and without GC at baseline, using a fixed‐effect model. (a) Forest plot of Atrophy without GC. (b) Forest plot of Atrophy with GC. GC: Gastric cancer.

**FIGURE S3** Forest plot analyzing the occurrence of post‐eradication gastric cancer according to the severity of intestinal metaplasia, using a fixed‐effect model. IM: intestinal metaplasia.

**FIGURE S4** Forest plot analyzing the occurrence of post‐eradication gastric cancer according to the post‐eradication onset of map‐like redness, using a fixed‐effect model. (a) Forest plot analyzed using all articles. (b) Forest plot analyzed using selected two prospective cohort studies.

**FIGURE S5** Forest plot analyzing the incidence of post‐eradication gastric cancer according to the appearance of xanthoma, using a fixed‐effect model.

**FIGURE S6** Funnel plots of atrophy analyzed, using a fixed‐effect model. (a) Atrophy of all 16 articles, (b) atrophy of seven articles without GC, and (c) atrophy of nine articles with GC.

**FIGURE S7** Funnel plots of intestinal metaplasia, map‐like redness, and xanthoma analyzed, using a fixed‐effect model. (a) Intestinal metaplasia, (b) map‐like redness of all six articles, (c) map‐like redness of selected two articles, and (d) xanthoma.

PRISMA2020 checklist.

## References

[1] Ferlay JEM , Lam F , Laversanne M *et al*. Global Cancer Observatory: Cancer Today, Lyon, France: International Agency for Research on Cancer [Internet], 2024, Available from: https://gco.iarc.who.int/today

[2] Ferlay J , Colombet M , Soerjomataram I *et al.* Estimating the global cancer incidence and mortality in 2018: GLOBOCAN sources and methods. Int J Cancer 2019; 144: 1941–1953. 30350310 10.1002/ijc.31937

[3] Uemura N , Okamoto S , Yamamoto S *et al.* *Helicobacter pylori* infection and the development of gastric cancer. N Engl J Med 2001; 345: 784–789. 11556297 10.1056/NEJMoa001999

[4] Herrero R , Park JY , Forman D . The fight against gastric cancer – The IARC Working Group report. Best Pract Res Clin Gastroenterol 2014; 28: 1107–1114. 25439075 10.1016/j.bpg.2014.10.003

[5] Take S , Mizuno M , Ishiki K *et al.* The effect of eradicating *Helicobacter pylori* on the development of gastric cancer in patients with peptic ulcer disease. Am J Gastroenterol 2005; 100: 1037–1042. 15842576 10.1111/j.1572-0241.2005.41384.x

[6] Fukase K , Kato M , Kikuchi S *et al.* Effect of eradication of *Helicobacter pylori* on incidence of metachronous gastric carcinoma after endoscopic resection of early gastric cancer: An open‐label, randomised controlled trial. Lancet 2008; 372: 392–397. 18675689 10.1016/S0140-6736(08)61159-9

[7] Ford AC , Forman D , Hunt RH , Yuan Y , Moayyedi P . *Helicobacter pylori* eradication therapy to prevent gastric cancer in healthy asymptomatic infected individuals: Systematic review and meta‐analysis of randomised controlled trials. BMJ 2014; 348: g3174. 24846275 10.1136/bmj.g3174 PMC4027797

[8] Yoon SB , Park JM , Lim CH , Cho YK , Choi MG . Effect of *Helicobacter pylori* eradication on metachronous gastric cancer after endoscopic resection of gastric tumors: A meta‐analysis. Helicobacter 2014; 19: 243–248. 25056262 10.1111/hel.12146

[9] Take S , Mizuno M , Ishiki K *et al.* Seventeen‐year effects of eradicating *Helicobacter pylori* on the prevention of gastric cancer in patients with peptic ulcer; A prospective cohort study. J Gastroenterol 2015; 50: 638–644. 25351555 10.1007/s00535-014-1004-5

[10] Wei Y , Min C , Zhao C *et al.* Endoscopic characteristics and high‐risk background mucosa factors of early gastric cancer after *Helicobacter pylori* eradication: A single‐center retrospective study. Front Oncol 2023; 13: 1272187. 37849804 10.3389/fonc.2023.1272187 PMC10577436

[11] Yamamoto K , Kato M , Takahashi M *et al.* Clinicopathological analysis of early‐stage gastric cancers detected after successful eradication of *Helicobacter pylori* . Helicobacter 2011; 16: 210–216. 21585606 10.1111/j.1523-5378.2011.00833.x

[12] Kodama M , Okimoto T , Mizukami K *et al.* Endoscopic and Immunohistochemical Characteristics of Gastric Cancer with versus without *Helicobacter pylori* Eradication. Digestion 2018; 97: 288–297. 29514141 10.1159/000485504

[13] Kobayashi M , Sato Y , Terai S . Endoscopic surveillance of gastric cancers after *Helicobacter pylori* eradication. World J Gastroenterol 2015; 21: 10553–10562. 26457015 10.3748/wjg.v21.i37.10553 PMC4588077

[14] Kitamura Y , Ito M , Matsuo T *et al.* Characteristic epithelium with low‐grade atypia appears on the surface of gastric cancer after successful *Helicobacter pylori* eradication therapy. Helicobacter 2014; 19: 289–295. 24766284 10.1111/hel.12132

[15] Masuda K , Urabe Y , Ito M *et al.* Genomic landscape of epithelium with low‐grade atypia on gastric cancer after *Helicobacter pylori* eradication therapy. J Gastroenterol 2019; 54: 907–915. 31197475 10.1007/s00535-019-01596-4 PMC6759680

[16] Ito M , Tanaka S , Takata S *et al.* Morphological changes in human gastric tumours after eradication therapy of *Helicobacter pylori* in a short‐term follow‐up. Aliment Pharmacol Ther 2005; 21: 559–566. 15740539 10.1111/j.1365-2036.2005.02360.x

[17] Kim N , Park YS , Cho SI *et al.* Prevalence and risk factors of atrophic gastritis and intestinal metaplasia in a Korean population without significant gastroduodenal disease. Helicobacter 2008; 13: 245–255. 18665932 10.1111/j.1523-5378.2008.00604.x

[18] Kodama M , Murakami K , Okimoto T *et al.* Histological characteristics of gastric mucosa prior to *Helicobacter pylori* eradication may predict gastric cancer. Scand J Gastroenterol 2013; 48: 1249–1256. 24079881 10.3109/00365521.2013.838994

[19] Kwon H , Lee SY , Kim JH *et al.* ABC Classification Is Less Useful for Older Koreans Born before 1960. Gut Liver 2019; 13: 522–530. 30970432 10.5009/gnl18399 PMC6743811

[20] Kodama M , Mizukami K , Hirashita Y *et al.* Differences in clinical features and morphology between differentiated and undifferentiated gastric cancer after *Helicobacter pylori* eradication. PLoS One 2023; 18: e0282341. 37000845 10.1371/journal.pone.0282341 PMC10065271

[21] Ford AC , Forman D , Hunt R , Yuan Y , Moayyedi P . *Helicobacter pylori* eradication for the prevention of gastric neoplasia. Cochrane Database Syst Rev 2015; 2015: CD005583. 26198377 10.1002/14651858.CD005583.pub2 PMC7263416

[22] Lee YC , Chiang TH , Chou CK *et al.* Association Between *Helicobacter pylori* eradication and gastric cancer incidence: A systematic review and meta‐analysis. Gastroenterology 2016; 150: 1113–1124.e5. 26836587 10.1053/j.gastro.2016.01.028

[23] Sugano K . Effect of *Helicobacter pylori* eradication on the incidence of gastric cancer: A systematic review and meta‐analysis. Gastric Cancer 2019; 22: 435–445. 30206731 10.1007/s10120-018-0876-0

[24] Sugimoto M , Murata M , Yamaoka Y . Chemoprevention of gastric cancer development after *Helicobacter pylori* eradication therapy in an East Asian population: Meta‐analysis. World J Gastroenterol 2020; 26: 1820–1840. 32351296 10.3748/wjg.v26.i15.1820 PMC7183870

[25] Dixon MF , Genta RM , Yardley JH , Correa P . Classification and grading of gastritis. The updated Sydney System. International Workshop on the Histopathology of Gastritis, Houston 1994. Am J Surg Pathol 1996; 20: 1161–1181. 8827022 10.1097/00000478-199610000-00001

[26] Kimura K , Takemoto T . An endoscopic recognition of the atrophic border and its significance in chronic gastritis. Endoscopy 1969; 1: 87–97.

[27] Haruma K . Kato M , Inoue K , Murakami K , Kamada T . Kyoto Classification of Gastritis, Tokyo: Nihon Medical Center, 2017.

[28] Uedo N , Ishihara R , Iishi H *et al.* A new method of diagnosing gastric intestinal metaplasia: Narrow‐band imaging with magnifying endoscopy. Endoscopy 2006; 38: 819–824. 17001572 10.1055/s-2006-944632

[29] Yao K , Iwashita A , Nambu M *et al.* Nature of white opaque substance in gastric epithelial neoplasia as visualized by magnifying endoscopy with narrow‐band imaging. Dig Endosc 2012; 24: 419–425. 23078433 10.1111/j.1443-1661.2012.01314.x

[30] Moribata K , Iguchi JK , Nakachi K *et al.* Endoscopic features associated with development of metachronous gastric cancer in patients who underwent endoscopic resection followed by *Helicobacter pylori* eradication. Dig Endosc 2016; 28: 434–442. 26623565 10.1111/den.12581

[31] Matsumoto S , Sugimoto M , Fukuzawa M *et al.* Risk of map‐like redness development after eradication therapy for *Helicobacter pylori* infection. Helicobacter 2024; 29: e13046. 38984721 10.1111/hel.13046

[32] Yan L , Chen Y , Chen F *et al.* Effect of *Helicobacter pylori* eradication on gastric cancer prevention: Updated report from a randomized controlled trial with 26.5 years of follow‐up. Gastroenterology 2022; 163: 154–162.e3. 35364066 10.1053/j.gastro.2022.03.039

[33] Choi JM , Kim SG , Choi J *et al.* Effects of *Helicobacter pylori* eradication for metachronous gastric cancer prevention: A randomized controlled trial. Gastrointest Endosc 2018; 88: 475–485 e2. 10.1016/j.gie.2018.05.009 29800546

[34] Ford AC , Yuan Y , Moayyedi P . *Helicobacter pylori* eradication therapy to prevent gastric cancer: Systematic review and meta‐analysis. Gut 2020; 69: 2113–2121. 32205420 10.1136/gutjnl-2020-320839

[35] Uemura N , Mukai T , Okamoto S *et al.* Effect of *Helicobacter pylori* eradication on subsequent development of cancer after endoscopic resection of early gastric cancer. Cancer Epidemiol Biomarkers Prev 1997; 6: 639–642. 9264278

[36] Wong BC , Lam SK , Wong WM *et al.* *Helicobacter pylori* eradication to prevent gastric cancer in a high‐risk region of China: A randomized controlled trial. JAMA 2004; 291: 187–194. 14722144 10.1001/jama.291.2.187

[37] Take S , Mizuno M , Ishiki K *et al.* Baseline gastric mucosal atrophy is a risk factor associated with the development of gastric cancer after *Helicobacter pylori* eradication therapy in patients with peptic ulcer diseases. J Gastroenterol 2007; 42 (Suppl 17): 21–27. 17238021 10.1007/s00535-006-1924-9

[38] Rokkas T , Sechopoulos P , Pistiolas D , Margantinis G , Koukoulis G . *Helicobacter pylori* infection and gastric histology in first‐degree relatives of gastric cancer patients: A meta‐analysis. Eur J Gastroenterol Hepatol 2010; 22: 1128–1133. 20410824 10.1097/MEG.0b013e3283398d37

[39] Kosunen TU , Pukkala E , Sarna S *et al.* Gastric cancers in Finnish patients after cure of *Helicobacter pylori* infection: A cohort study. Int J Cancer 2011; 128: 433–439. 20309944 10.1002/ijc.25337

[40] Yan X , Hu X , Duan B *et al.* Exploration of endoscopic findings and risk factors of early gastric cancer after eradication of *Helicobacter pylori* . Scand J Gastroenterol 2021; 56: 356–362. 33410344 10.1080/00365521.2020.1868567

[41] Shibukawa N , Ouchi S , Wakamatsu S , Wakahara Y , Kaneko A . Gastric xanthoma is a predictive marker for early gastric cancer detected after *Helicobacter pylori* eradication. Intern Med 2019; 58: 779–784. 30449773 10.2169/internalmedicine.0925-18 PMC6465014

[42] Hara D , Okamura T , Iwaya Y , Nagaya T , Ota H , Umemura T . Histopathologically defined intestinal metaplasia in lesser curvature of corpus prior to *Helicobacter pylori* eradication is a risk factor for gastric cancer development. Helicobacter 2022; 27: e12934. 36263778 10.1111/hel.12934

[43] Shichijo S , Hirata Y . Characteristics and predictors of gastric cancer after *Helicobacter pylori* eradication. World J Gastroenterol 2018; 24: 2163–2172. 29853734 10.3748/wjg.v24.i20.2163 PMC5974578

[44] Azuma T . *Helicobacter pylori* CagA protein variation associated with gastric cancer in Asia. J Gastroenterol 2004; 39: 97–103. 15069615 10.1007/s00535-003-1279-4

[45] Yamaoka Y . Mechanisms of disease: *Helicobacter pylori* virulence factors. Nat Rev Gastroenterol Hepatol 2010; 7: 629–641. 20938460 10.1038/nrgastro.2010.154 PMC3137895

[46] Take S , Mizuno M , Ishiki K *et al.* The long‐term risk of gastric cancer after the successful eradication of *Helicobacter pylori* . J Gastroenterol 2011; 46: 318–324. 21103997 10.1007/s00535-010-0347-9

[47] Liu Y , Vosmaer GD , Tytgat GN , Xiao SD , Ten Kate FJ . Gastrin (G) cells and somatostatin (D) cells in patients with dyspeptic symptoms: *Helicobacter pylori* associated and non‐associated gastritis. J Clin Pathol 2005; 58: 927–931. 16126872 10.1136/jcp.2003.010710 PMC1770830

[48] Quach DT , Le HM , Hiyama T , Nguyen OT , Nguyen TS , Uemura N . Relationship between endoscopic and histologic gastric atrophy and intestinal metaplasia. Helicobacter 2013; 18: 151–157. 23167960 10.1111/hel.12027

[49] Kodama M , Okimoto T , Ogawa R , Mizukami K , Murakami K . Endoscopic atrophic classification before and after *H. pylori* eradication is closely associated with histological atrophy and intestinal metaplasia. Endosc Int Open 2015; 3: E311–E317. 26357676 10.1055/s-0034-1392090 PMC4554494

[50] Kodama M , Murakami K , Okimoto T *et al.* Ten‐year prospective follow‐up of histological changes at five points on the gastric mucosa as recommended by the updated Sydney system after *Helicobacter pylori* eradication. J Gastroenterol 2012; 47: 394–403. 22138891 10.1007/s00535-011-0504-9

[51] Rokkas T , Rokka A , Portincasa P . A systematic review and meta‐analysis of the role of *Helicobacter pylori* eradication in preventing gastric cancer. Ann Gastroenterol 2017; 30: 414–423. 28655977 10.20524/aog.2017.0144 PMC5479993

[52] Kong YJ , Yi HG , Dai JC , Wei MX . Histological changes of gastric mucosa after *Helicobacter pylori* eradication: A systematic review and meta‐analysis. World J Gastroenterol 2014; 20: 5903–5911. 24914352 10.3748/wjg.v20.i19.5903 PMC4024801

[53] Hwang YJ , Kim N , Lee HS *et al.* Reversibility of atrophic gastritis and intestinal metaplasia after *Helicobacter pylori* eradication – A prospective study for up to 10 years. Aliment Pharmacol Ther 2018; 47: 380–390. 29193217 10.1111/apt.14424

[54] Fukuda K , Kodama M , Mizukami K *et al.* Analysis of long‐term serological and histological changes after eradication of *Helicobacter pylori* . J Clin Biochem Nutr 2022; 71: 151–157. 36213784 10.3164/jcbn.21-164 PMC9519420

[55] Shichijo S , Hirata Y , Niikura R *et al.* Histologic intestinal metaplasia and endoscopic atrophy are predictors of gastric cancer development after *Helicobacter pylori* eradication. Gastrointest Endosc 2016; 84: 618–624. 26995689 10.1016/j.gie.2016.03.791

[56] Choi IJ , Kook MC , Kim YI *et al.* *Helicobacter pylori* therapy for the prevention of metachronous gastric cancer. N Engl J Med 2018; 378: 1085–1095. 29562147 10.1056/NEJMoa1708423

[57] Rokkas T , Pistiolas D , Sechopoulos P , Robotis I , Margantinis G . The long‐term impact of *Helicobacter pylori* eradication on gastric histology: A systematic review and meta‐analysis. Helicobacter 2007; 12 (Suppl 2): 32–38. 17991174 10.1111/j.1523-5378.2007.00563.x

[58] Wang J , Xu L , Shi R *et al.* Gastric atrophy and intestinal metaplasia before and after *Helicobacter pylori* eradication: A meta‐analysis. Digestion 2011; 83: 253–260. 21282951 10.1159/000280318

[59] Takeuchi C , Yamashita S , Liu YY *et al.* Precancerous nature of intestinal metaplasia with increased chance of conversion and accelerated DNA methylation. Gut 2024; 73: 255–267. 37751933 10.1136/gutjnl-2023-329492

[60] Majima A , Dohi O , Takayama S *et al.* Linked color imaging identifies important risk factors associated with gastric cancer after successful eradication of *Helicobacter pylori* . Gastrointest Endosc 2019; 90: 763–769. 31299258 10.1016/j.gie.2019.06.043

[61] Take S , Mizuno M , Ishiki K *et al.* Risk of gastric cancer in the second decade of follow‐up after *Helicobacter pylori* eradication. J Gastroenterol 2020; 55: 281–288. 31667586 10.1007/s00535-019-01639-w PMC7026240

[62] Kobayashi M , Fujisaki J , Namikawa K *et al.* Multicenter study of invasive gastric cancer detected after 10 years of *Helicobacter pylori* eradication in Japan: Clinical, endoscopic, and histopathologic characteristics. DEN Open 2024; 4: e345. 38434145 10.1002/deo2.345 PMC10908369

[63] Kamada T , Hata J , Sugiu K *et al.* Clinical features of gastric cancer discovered after successful eradication of *Helicobacter pylori*: Results from a 9‐year prospective follow‐up study in Japan. Aliment Pharmacol Ther 2005; 21: 1121–1126. 15854174 10.1111/j.1365-2036.2005.02459.x

[64] Kwon YH , Heo J , Lee HS , Cho CM , Jeon SW . Failure of *Helicobacter pylori* eradication and age are independent risk factors for recurrent neoplasia after endoscopic resection of early gastric cancer in 283 patients. Aliment Pharmacol Ther 2014; 39: 609–618. 24461252 10.1111/apt.12633

[65] Toyoshima O , Yamaji Y , Yoshida S *et al.* Endoscopic gastric atrophy is strongly associated with gastric cancer development after *Helicobacter pylori* eradication. Surg Endosc 2017; 31: 2140–2148. 27604367 10.1007/s00464-016-5211-4 PMC5411409

[66] Kato M , Hayashi Y , Nishida T *et al.* *Helicobacter pylori* eradication prevents secondary gastric cancer in patients with mild‐to‐moderate atrophic gastritis. J Gastroenterol Hepatol 2021; 36: 2083–2090. 33403702 10.1111/jgh.15396

[67] Hanaoka N , Uedo N , Shiotani A *et al.* Autofluorescence imaging for predicting development of metachronous gastric cancer after *Helicobacter pylori* eradication. J Gastroenterol Hepatol 2010; 25: 1844–1849. 21091995 10.1111/j.1440-1746.2010.06442.x

[68] Maehata Y , Nakamura S , Fujisawa K *et al.* Long‐term effect of *Helicobacter pylori* eradication on the development of metachronous gastric cancer after endoscopic resection of early gastric cancer. Gastrointest Endosc 2012; 75: 39–46. 22018552 10.1016/j.gie.2011.08.030

[69] Take S , Mizuno M , Ishiki K *et al.* Correction to: Risk of gastric cancer in the second decade of follow‐up after *Helicobacter pylori* eradication. J Gastroenterol 2020; 55: 289–290. 31820091 10.1007/s00535-019-01654-x PMC7645480

[70] Ohno A , Miyoshi J , Kato A *et al.* Endoscopic severe mucosal atrophy indicates the presence of gastric cancer after *Helicobacter pylori* eradication ‐analysis based on the Kyoto classification. BMC Gastroenterol 2020; 20: 232. 32689949 10.1186/s12876-020-01375-z PMC7370417

[71] Liu X , Wang X , Mao T *et al.* Characteristic analysis of early gastric cancer after *Helicobacter pylori* eradication: A multicenter retrospective propensity score‐matched study. Ann Med 2023; 55: 2231852. 37450336 10.1080/07853890.2023.2231852 PMC10351464

