# Supplementary figures and images for: Endoscopic risk factors to inform early detection of gastric cancer after Helicobacter pylori eradication: Meta‐analysis and systematic review
Source: DEN Open. 2025 Feb 26;5(1):e70086. doi: 10.1002/deo2.70086 (PMC11865013; doi:10.1002/deo2.70086)

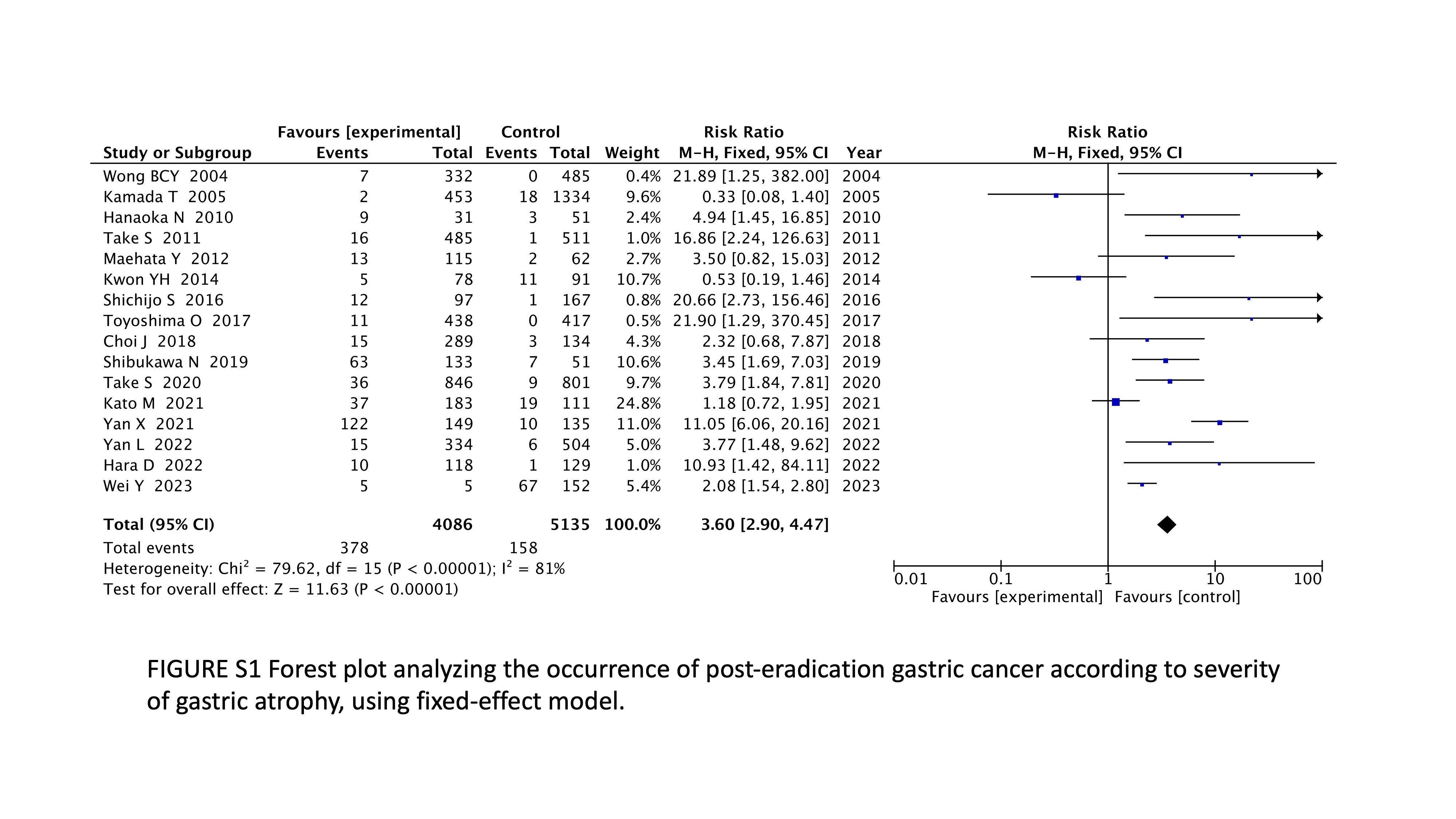

Supplement: Supplementary file 1 — FIGURE S1 Forest plot analyzing the occurrence of post‐eradication gastric cancer according to the severity of gastric atrophy, using a fixed‐effect model. [file DEO2-5-e70086-s002.jpg]

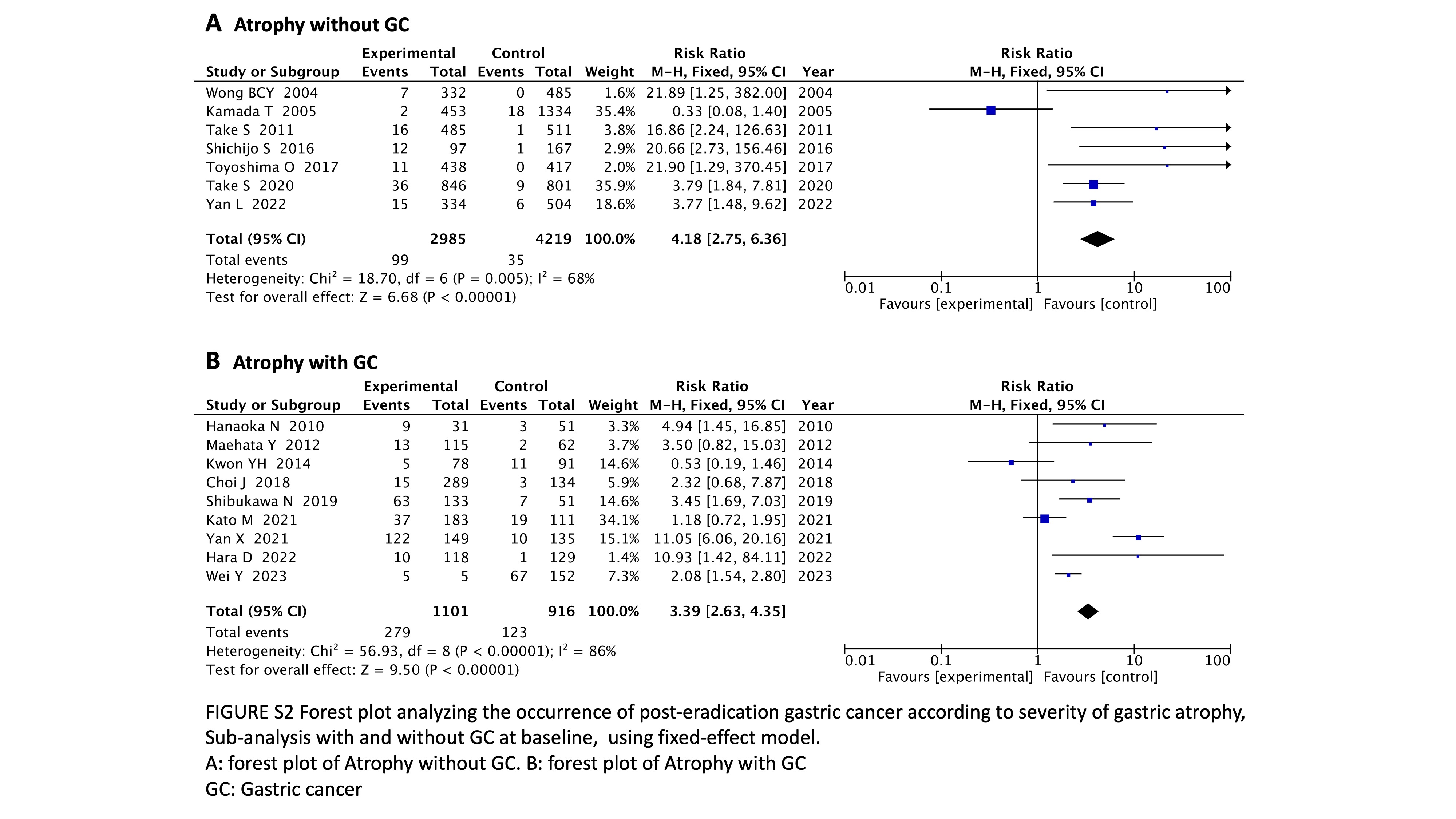

Supplement: Supplementary file 2 — FIGURE S2 Forest plot analyzing the occurrence of post‐eradication gastric cancer according to the severity of gastric atrophy, Sub‐analysis with and without GC at baseline, using a fixed‐effect model. (a) Forest plot of Atrophy without GC. (b) Forest plot of Atrophy with GC. GC: Gastric cancer. [file DEO2-5-e70086-s001.jpg]

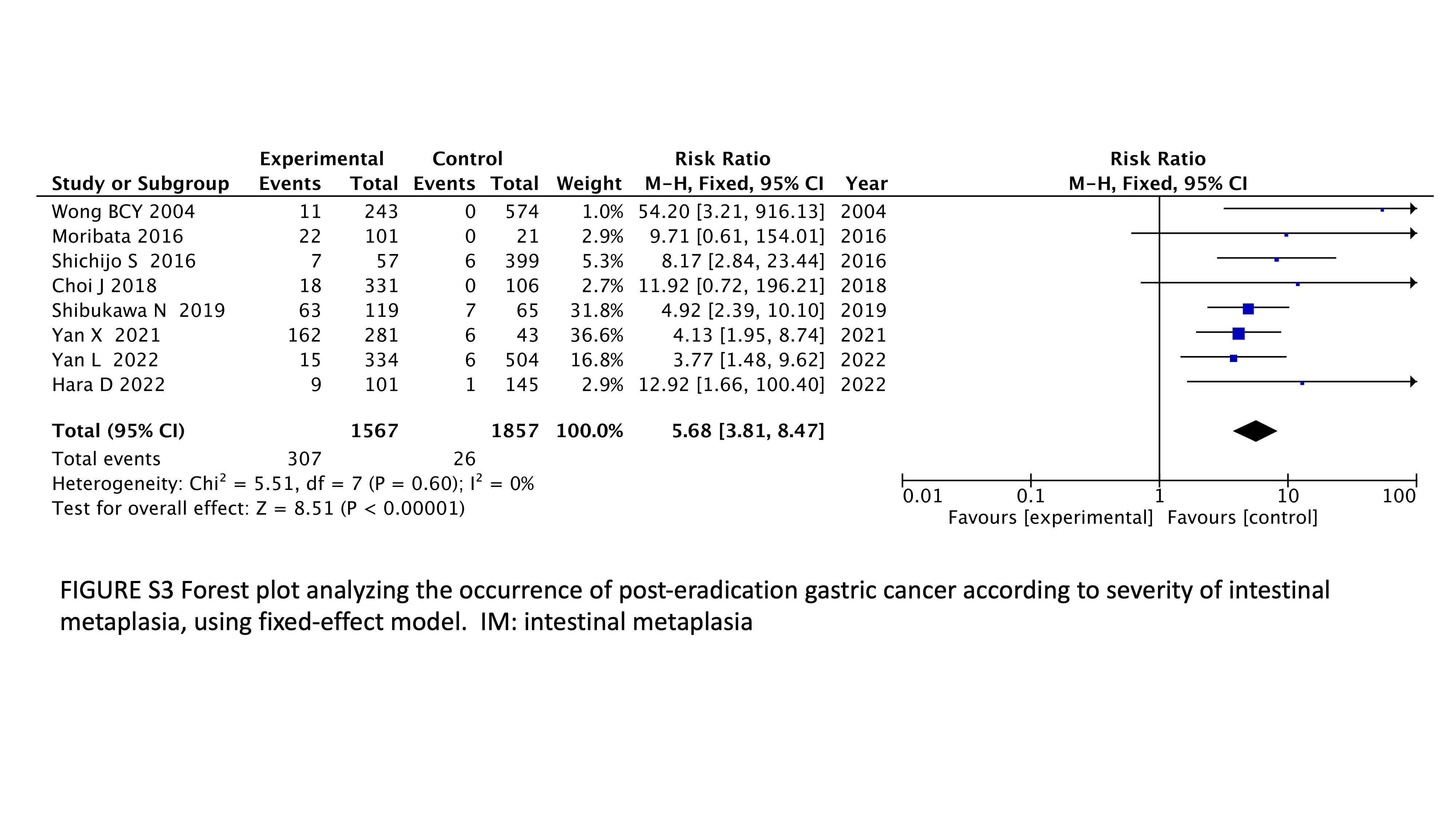

Supplement: Supplementary file 3 — FIGURE S3 Forest plot analyzing the occurrence of post‐eradication gastric cancer according to the severity of intestinal metaplasia, using a fixed‐effect model. IM: intestinal metaplasia. [file DEO2-5-e70086-s005.jpg]

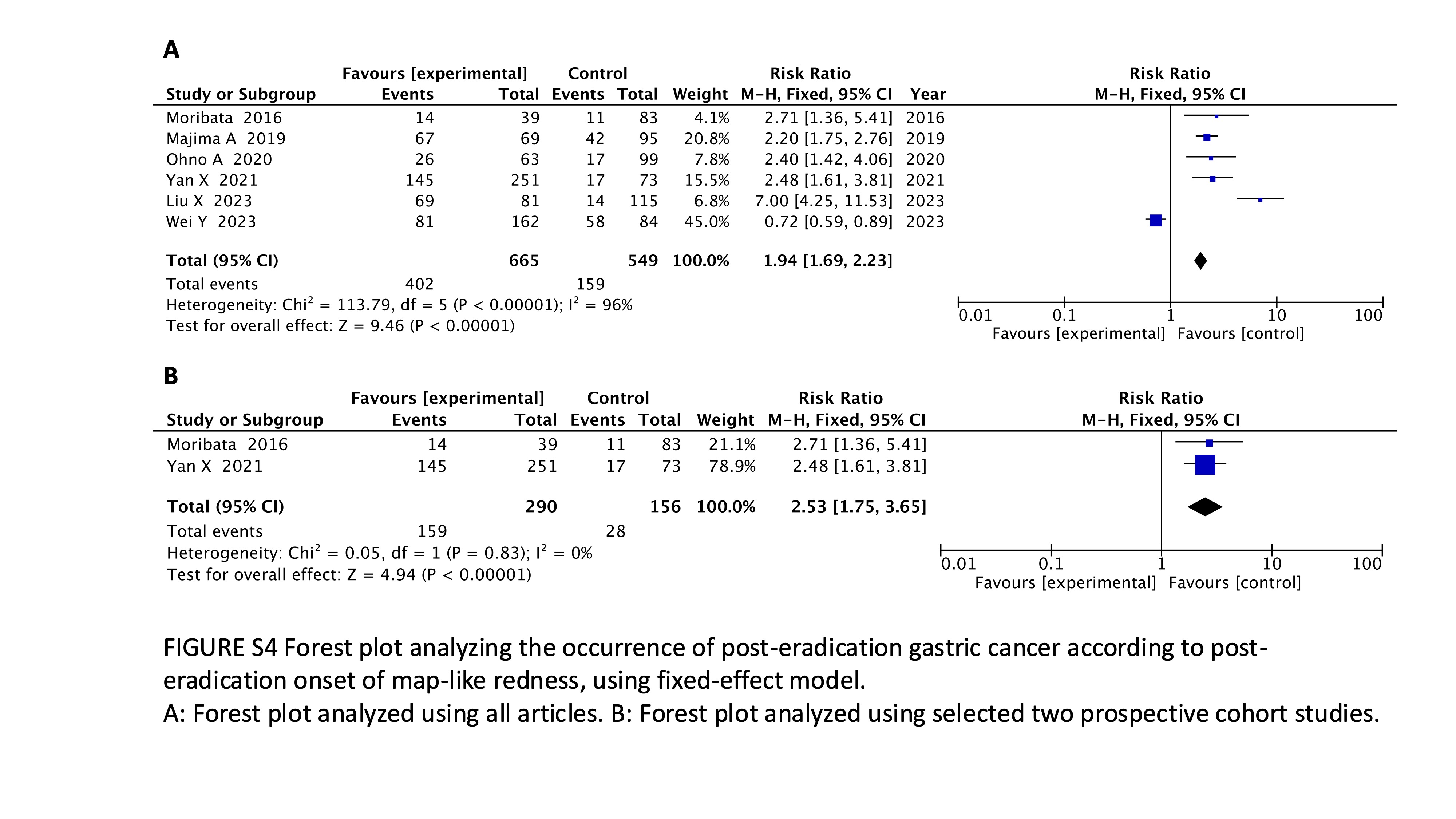

Supplement: Supplementary file 4 — FIGURE S4 Forest plot analyzing the occurrence of post‐eradication gastric cancer according to the post‐eradication onset of map‐like redness, using a fixed‐effect model. (a) Forest plot analyzed using all articles. (b) Forest plot analyzed using selected two prospective cohort studies. [file DEO2-5-e70086-s006.jpg]

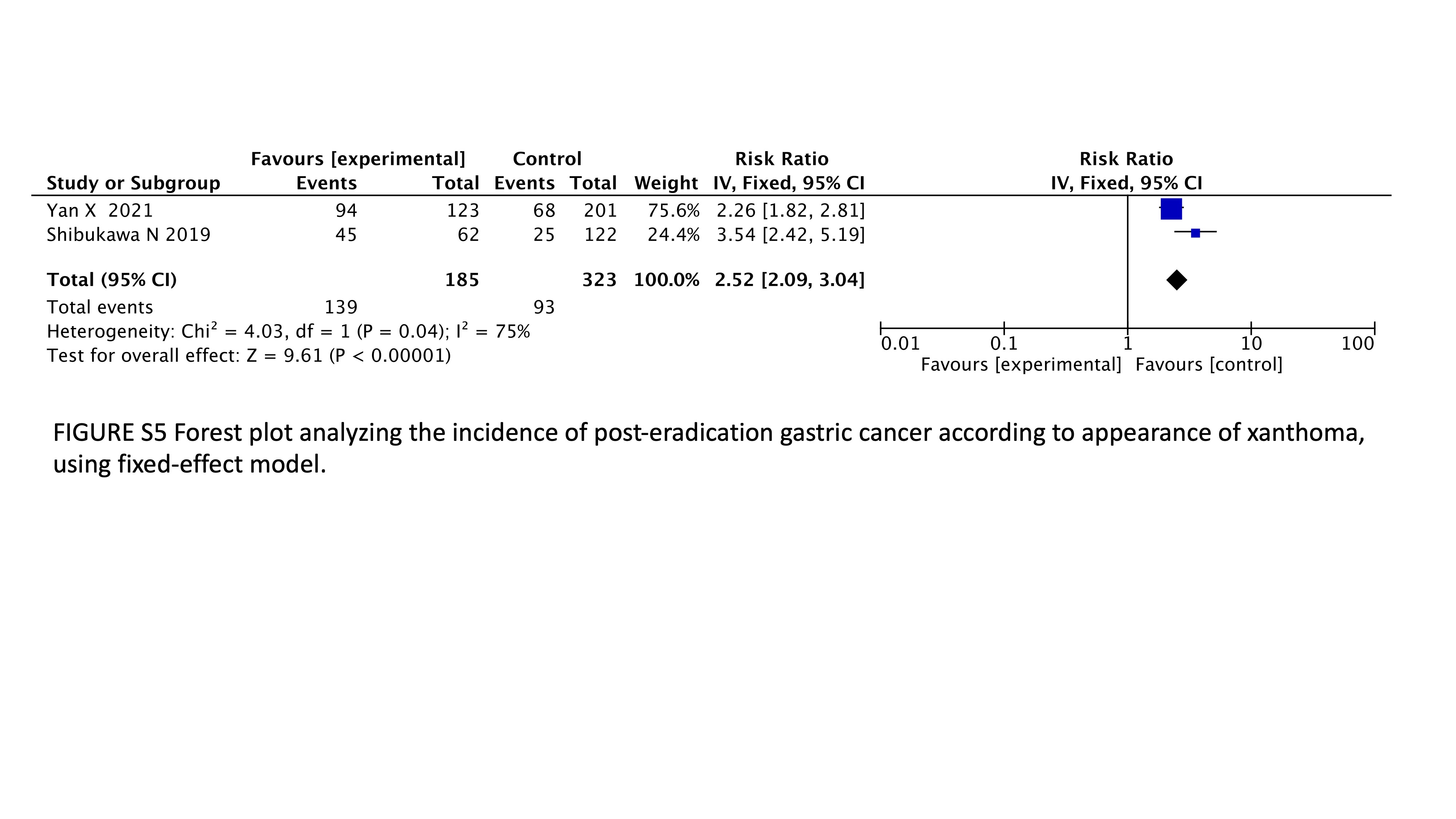

Supplement: Supplementary file 5 — FIGURE S5 Forest plot analyzing the incidence of post‐eradication gastric cancer according to the appearance of xanthoma, using a fixed‐effect model. [file DEO2-5-e70086-s008.jpg]

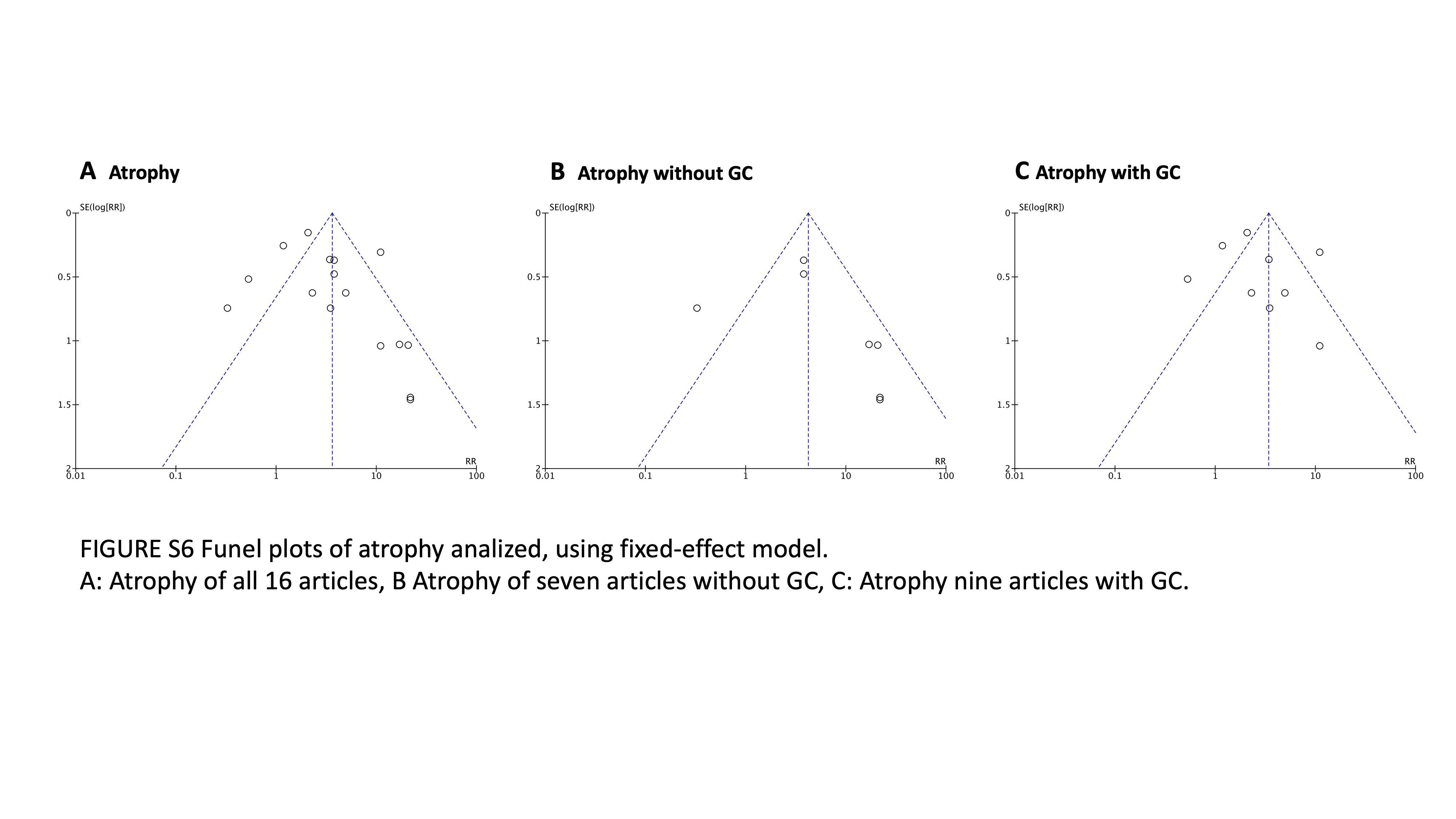

Supplement: Supplementary file 6 — FIGURE S6 Funnel plots of atrophy analyzed, using a fixed‐effect model. (a) Atrophy of all 16 articles, (b) atrophy of seven articles without GC, and (c) atrophy of nine articles with GC. [file DEO2-5-e70086-s004.jpg]

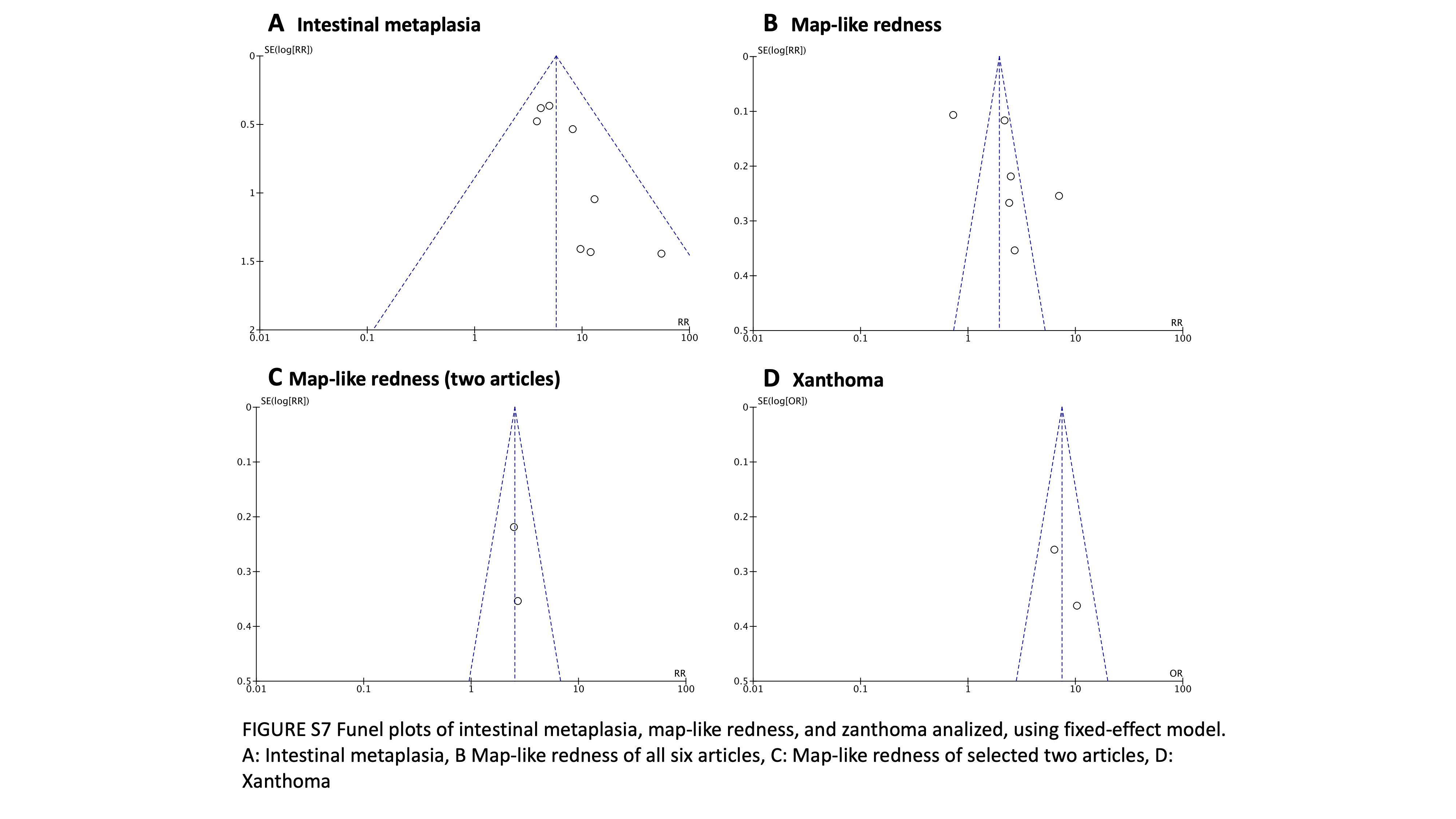

Supplement: Supplementary file 7 — FIGURE S7 Funnel plots of intestinal metaplasia, map‐like redness, and xanthoma analyzed, using a fixed‐effect model. (a) Intestinal metaplasia, (b) map‐like redness of all six articles, (c) map‐like redness of selected two articles, and (d) xanthoma. [file DEO2-5-e70086-s007.jpg]
